# Performance Anxiety and Academic Performance: Academic, Physical, and Psychological Effects in Students of Higher Studies in Music

**DOI:** 10.3390/bs15111532

**Published:** 2025-11-10

**Authors:** Paula Hernández-Dionis, David Pérez-Jorge, Pablo J. Borges-Hernández, Anthea Gara Santos-Álvarez

**Affiliations:** Facultad de Educación, Universidad de La Laguna, Calle Padre Herrera, s/n, 38200 La Laguna, Santa Cruz de Tenerife, Spain; phernadi@ull.edu.es (P.H.-D.); pborgesh@ull.edu.es (P.J.B.-H.); asantosa@ull.edu.es (A.G.S.-Á.)

**Keywords:** musical stage anxiety, self-confidence, conservatory, pedagogy

## Abstract

Musical performance anxiety (MPA) is a common phenomenon among conservatory students that has direct effects on their academic performance and psychological well-being. This study examines the impact of MPA on students at the Conservatorio Superior de Música de Canarias, employing a multidimensional approach that integrates psychological variables, coping strategies, physical activity, and entertainment habits. The K-MPAI, DPAA, and MLTPAQ questionnaires were applied using descriptive analysis, nonparametric tests, correlations, and a mediation model. Cognitive anxiety was identified as the main component of MPA, while self-confidence acted as a protective and mediating factor against motivation. Visualization was associated with motivation and coping with differences by specialty (pedagogy and interpretation), and no links were found between physical activity and MPA. The results indicate the need for MPA to be addressed comprehensively, incorporating technical training, psychological support, and wellness strategies.

## 1. Introduction

Musical performance anxiety (MPA) manifests as an adverse emotional reaction to the prospect of performing in public, generating physiological, cognitive, and behavioral symptoms that directly affect performance (Papageorgi et al., 2011). MPA is generally conceptualized as a multidimensional construct encompassing cognitive, emotional, and physiological responses to performance-related stress (Kenny, 2011). Somatic anxiety, one of its core components, refers to the physiological manifestations of this state, such as increased heart rate, trembling, or muscle tension, that can interfere with performance execution (Osborne & Kenny, 2008). In this context, MPA not only impairs the quality of performances but also influences the formative experience, eventually limiting musicians’ professional development (Fernholz et al., 2019; Yang et al., 2025; Wang & Yang, 2024).

Music students with high levels of anxiety often avoid public performance opportunities, which restricts their learning and artistic development (Dalia, 2004; Jadue, 2001; Urruzola & Bernaras, 2019). It has been observed that high levels of self-demand and fear of negative judgment can amplify this condition, making musicians more susceptible to experiencing chronic stage stress (Butković et al., 2021; Patston & Osborne, 2016).

Self-efficacy emerges as a crucial factor in mitigating stage anxiety in the musical context (Casanova et al., 2023). In this study, self-efficacy is understood as students’ belief in their own capacity to successfully perform musical tasks under pressure, following Bandura’s social cognitive framework (McPherson & McCormick, 2006). The ability to overcome challenges enables students to face high-pressure situations, such as auditions and concerts, with greater confidence (McPherson & McCormick, 2006). Self-efficacy is also associated with greater intrinsic motivation and the adoption of effective strategies for coping with the demands of music study (Ritchie & Williamon, 2011).

Self-esteem is defined as the individual’s overall evaluation of their own worth and competence, which, in the musical context, is shaped by performance experiences and social feedback. An aspect closely linked to MPA and self-efficacy is self-esteem, which develops as a function of perceptions of achievement, support from teachers and peers, and feedback on performance (Kenny, 2011; Plummer, 2001; Ramos, 2013). The construction of positive self-esteem can act as a buffer against anxiety, while negative self-worth increases emotional vulnerability, affecting students’ well-being and artistic performance (Martínez-Antón et al., 2007; Rodriguez et al., 2023). These connections highlight the importance of addressing the psychological dimension in music training programs, promoting strategies that enhance personal confidence and mitigate risk factors associated with MPA (Jiang, 2024; Nwokenna et al., 2022). These variables not only modulate MPA but are also directly related to academic performance, as they condition students’ ability to display their competencies in public assessments (Montiel & Clares-Clares, 2023; Orejudo et al., 2021; Zarza-Alzugaray et al., 2020).

MPA can directly impact academic performance in the musical context, particularly when students are evaluated on their public performances (Biasutti & Concina, 2014; Wilson & Roland, 2002). The presence of symptoms such as tremors, mental blocking, or catastrophic thinking can lead the student to fail to demonstrate their actual level of competence, negatively impacting their grades and self-efficacy (Osborne & Kenny, 2008).

Structured preparation, practice in simulated performance conditions, and the incorporation of emotional regulation techniques (such as conscious breathing or positive visualization) can significantly decrease anxiety levels (Araújo et al., 2017; Fernholz et al., 2019). Adequate preparation encompasses not only technical and interpretive aspects, but also psychological strategies that reinforce the musician’s confidence, allowing them to face high-pressure situations with greater emotional stability (Braden et al., 2015; Finch & Moscovitch, 2016). Visualization has been identified as a specific cognitive strategy, whereby the student mentally anticipates the performance. Its use is associated with reduced MPA and improved academic performance on music assessments (Brown & Palmer, 2013; Esplen & Hodnett, 1999).

The family and emotional environment play a critical role in shaping music students’ MPA. Early childhood experiences, attachment style, and the quality of emotional support received have lasting effects on self-efficacy and emotional response to public judgment (Osborne & Franklin, 2002; Kenny, 2011). When parents are overly critical, controlling, or exhibit high levels of anxiety, students are more likely to develop dysfunctional cognitive schemas and fear of failure (Kirsner et al., 2023; Spence & Rapee, 2016).

Conversely, a family environment characterized by warmth, emotional acceptance, and open communication offers strong self-esteem and increased resilience in the face of stress (Papageorgi et al., 2011). Support from families, teachers, and peers promotes emotional self-efficacy and significantly reduces MPA during music performance (Huawei & Salarzadeh, 2024; Schneider & Chesky, 2011). In this sense, the family climate not only provides affective security but also practical resources, such as adequate spaces for instrumental practice, structured schedules, and emotional support in the face of academic demands, all of which are key to preventing MPA (Conger et al., 1994).

### 1.1. Psychological Determinants of Artistic Interpretation

Key psychological determinants linked to successful musical performance include aspects such as self-efficacy, attention, motivation, and causal attributions (McPherson & McCormick, 2006; Papageorgi et al., 2007). Self-efficacy in the musical context, understood as the belief in one’s own ability to perform a task successfully, has been shown to exert a protective effect against MPA (Zarza-Alzugaray et al., 2020; Huawei & Salarzadeh, 2024). Family, teacher, and peer support strengthen self-efficacy and prevent elevated MPA levels during public presentations (Burin & Osorio, 2016; Miksza & Tan, 2015). 

Students who report higher perceived family and teacher support in preservice show significantly lower levels of MPA, reinforcing the idea that self-efficacy mediates the relationship between environment and academic performance (Okan & Usta, 2021; Orejudo et al., 2021). In high-level evaluation and performance situations, locus of control (the way a person explains the causes of his or her successes or failures) plays vital role in a student’s relationship with the MPA. Motivation and locus of control are related to persistence, study quality, and academic performance (Evans & Bonneville-Roussy, 2015; Özen Kutanis et al., 2011). When outcomes are thought to depend primarily on one’s own effort and preparation, better interpretive performance and lower vulnerability to MPA are often observed (Nogaj, 2017). When what happens is attributed to luck or outside factors, it reduces the sense of personal control and increases anxiety levels (Lefcourt, 2014; Wolk & Bloom, 1978).

Students with a reduced ability to focus during performance often exhibit more intense MSA symptoms, including blocking, distractions by external stimuli, or difficulty retrieving musical memories (Burin & Osorio, 2016; Lupiáñez et al., 2021). Students’ intrinsic motivation and artistic enjoyment reduce stress before performance (Creech & Hallam, 2011; Mazzarolo et al., 2023; Ryan & Deci, 2000). Although anxiety can be facilitative at moderate levels, with high motivation, it can even enhance performance (Kenny, 2011; Osborne & Kenny, 2005). However, high levels of motivation, when not accompanied by sufficient self-confidence, can intensify MPA, as the student perceives greater internal pressure to perform with excellence (McPherson & McCormick, 2006; Ou & Qin, 2025). This is why maintaining a passion for music and constant training is considered crucial in the balanced development of the performer (Creech & Hallam, 2011).

Interpretive successes associated with preparation and effort favor the consolidation of self-efficacy (Bandura, 1997), while those attributed to luck or the teacher’s judgment reinforce feelings of incompetence and dependence (Pajares, 1996). This pattern underscores the importance of didactic strategies that enable students to understand their achievements from within, thereby enhancing their emotional and technical autonomy.

Teachers should not only be technical transmitters, but also facilitators of students’ psychological development (Creech & Hallam, 2011; Mazzarolo et al., 2023). Although teachers often focus on mastery experiences and verbal persuasion, in many cases, they lack specific training to work on students’ psychological skills, such as MPA control and emotional regulation (Kenny, 2011; Mazzarolo et al., 2023; Papageorgi et al., 2007). The need to incorporate training in coping techniques in music didactics is emphasized: relaxation, stage simulation, emotional management modeling, and constructive feedback (Kenny, 2023; Osborne & Franklin, 2002; Shaw et al., 2020).

### 1.2. Physical Activity and Its Impact on the MPA

Physical activity (PA) plays a crucial role in managing MPA, as it contributes to students’ overall well-being and serves a mechanism for emotional regulation (Biddle & Asare, 2011; Herbert et al., 2020; Mikkelsen et al., 2017; Salmon, 2001). In this study, PA is operationalized as any bodily movement that requires energy expenditure and contributes to physical fitness, including both moderate (e.g., walking, stretching) and vigorous activities (e.g., running, strength training). Participation in moderate and vigorous PA not only improves physical health but also has significant psychological benefits, such as reduced levels of stress and anxiety (Craft & Perna, 2004; Mikkelsen et al., 2017; Petruzzello et al., 1991). These activities may help counteract the adverse effects of accumulated stage stress on students in higher music studies.

Moderate PA, such as walking or stretching exercises, can help relieve muscle tension derived from long sessions of instrumental practice, facilitating greater physical and mental relaxation (Neves et al., 2014; Stults-Kolehmainen & Sinha, 2014; Tovar, 2018; Youngstedt et al., 1998). Practices such as yoga and meditation are effective in reducing MPA as they improve body awareness and promote controlled breathing (Cramer et al., 2018; Khalsa et al., 2009; Olabe, 2020; Palma-León et al., 2022).

Vigorous PA, such as running or strength training, contributes to emotional regulation as it increases the release of endorphins, improving mood and reducing anxiety symptoms (Anderson & Shivakumar, 2013). For music students, developing adequate physical endurance is also important as it allows them to maintain optimal energy levels during performances and better cope with the physical demands of instrumental learning (Araújo et al., 2020; Chan et al., 2013).

The context in which students perform PA also influences its effectiveness in reducing MPA (Biddle & Asare, 2011; Huang et al., 2024; Wipfli et al., 2008). The availability of adequate facilities for artistic practice and having sufficient time to incorporate exercise into the academic routine are determining factors (Lee et al., 2016; Reed, 2007; Sallis et al., 2016). Higher conservatories that promote programs that integrate PA and music training contribute to a more balanced and healthy educational environment for students (S. Donnelly et al., 2024; Huang et al., 2024; Teuber et al., 2024). This holistic approach not only addresses the physical dimension of students but also has a positive impact on their psychological well-being, mitigating the effects of MPA (Singh et al., 2023). 

Students’ entertainment habits significantly influence their emotional balance and coping capacity in the face of MPA (De Filippis & Al Foysal, 2025; Lemola et al., 2015). Excessive use of passive audiovisual media can negatively impact concentration, study time, and sleep quality, leading to increased emotional reactivity and anxious symptomatology (Hale & Guan, 2015; Twenge & Campbell, 2018). Active music listening, drawing, or playing active video games that involve physical movement can induce positive emotional states and improve affective self-regulation (Benedek et al., 2014; Granic et al., 2014). The balance between PA, rest, and healthy entertainment significantly improves the perception of well-being in musicians (Matei & Ginsborg, 2020; Terry et al., 2020). The positive impact of PA on emotional and physical well-being may also positively influence academic performance by promoting better rest, greater physical endurance during performances, and an increased ability to concentrate (Álvarez-Bueno et al., 2017; J. E. Donnelly et al., 2016; Shoebridge & Osborne, 2025).

Despite the growing literature on musical performance anxiety (MPA), few studies have jointly examined its relationship with psychological resources and physical activity among higher music education students. Understanding how these variables interact can inform the design of pedagogical and institutional strategies to promote students’ emotional well-being and academic performance. Therefore, this study seeks to fill this gap by analyzing the influence of self-efficacy, self-esteem, and physical activity on MPA within the context of the Conservatorio Superior de Música de Canarias, offering practical implications for music educators.

## 2. Objectives

The general objective of this study is to analyze the influence of psychological resources and PA on the MPA of students at the Conservatorio Superior de Música de Canarias (CSMC), considering their potential impact on academic performance and the modulating role of self-confidence. It is through this general objective that the following specific objectives are established:

SO1: To describe the levels of MPA (cognitive and somatic) in music students and their possible impact on academic performance.

SO2: To examine the influence of self-confidence as a modulating variable in the relationship between motivation and MPA, and its link with academic performance.

SO3: To compare the psychological dimensions assessed as a function of gender, grade, and musical specialty.

SO4: To analyze the relationship between PA practice and MPA levels, as well as its indirect impact on academic performance.

SO5: To identify psychological strategies with potential to improve both MPA management and academic performance in advanced training contexts.

## 3. Methodology

This non-experimental, cross-sectional quantitative study was designed to analyze quantifiable data and gain a better understanding of the phenomenon under investigation (Ulloa et al., 2020). The methodology directly linked the study objectives with the data collected through two standardized questionnaires.

### 3.1. Population and Sample

The population of this study is constituted by the students of the Conservatorio Superior de Música de Canarias in the specialties of Interpretation and Pedagogy.

The sample consisted of a total of 56 students from the Conservatorio Superior de Música de Canarias, located in its two campuses (Santa Cruz de Tenerife and Las Palmas de Gran Canaria). The participating specialties were Performance (n = 40) and Pedagogy (n = 16). As shown in Table 1, the total of 56 students comprised 32 men, 23 women, and one person who preferred not to specify (See Table 1).

### 3.2. Procedure

The questionnaires were selected based on the study’s objectives, and the proposal was evaluated and approved by the University of La Laguna’s Ethics Committee. Subsequently, the instruments, presented through the Google Form platform, were made available in person at the two sites of the Conservatorio Superior de Música de Canarias via a QR code or an online link, and each participant completed them using their own electronic device (cell phone or computer). Participation was voluntary, and data collection was conducted to maximize the response rate.

This study received the approval of the Research Ethics and Animal Welfare Committee (CEIBA) of the University of La Laguna, with registration number CEIBA2024-3481 (Approved on 29 September 2024). The study was conducted in accordance with the principles of the Declaration of Helsinki. All participants received detailed information and gave prior informed consent. Statistical analyses were performed with anonymized data to ensure privacy and confidentiality. The data were managed in accordance with the Organic Law 3/2018, of December 5, on Personal Data Protection and Guarantee of Digital Rights.

### 3.3. Instruments

Students completed the *Kenny Music Performance Anxiety Inventory* (K-MPAI) (Kenny, 2009), a 40-item instrument rated on a 7-point Likert scale (1 = strongly disagree to 7 = strongly agree) designed to comprehensively assess musical performance anxiety across cognitive, emotional, physiological, and personal background dimensions. The K-MPAI is one of the most widely used and internationally validated tools for studying performance anxiety in musicians, showing excellent internal consistency in different cultural contexts (α = 0.94 in the original version, (Kenny, 2009); α = 0.866 in the Spanish version, (Zarza-Alzugaray et al., 2020)). In the present study, the instrument also demonstrated high internal reliability (α = 0.916), confirming the internal coherence of the questionnaire within the analyzed sample.

The *Psychological Determinants for Successful Artistic Performance and Useful Rehearsals* (DPAA) is a 30-item questionnaire designed to assess various psychological variables related to artistic performance, such as self-confidence, motivation, locus of control, coping strategies, and emotional regulation. Its purpose is to identify personal strengths and limitations that may influence stage performance. Although no scientific publications have been found reporting the psychometric validation of the DPAA, the instrument has been cited and used in educational and performance psychology contexts in Spain. In the present study, internal consistency was empirically estimated from the collected data, yielding a Cronbach’s alpha of 0.67 for the total scale. This value reflects a moderate level of reliability, considered acceptable given the multidimensional nature of the construct assessed.

The *Minnesota Leisure Time Physical Activity Questionnaire* (MLTPAQ) (Taylor et al., 1978) is a self-administered instrument that assesses the amount and intensity of physical activity performed during leisure time over the previous week. The Spanish adaptation has demonstrated good validity and reliability, with test–retest intraclass correlation coefficients ranging from 0.35 to 0.62 (Elosua et al., 1994) and high concordance in its reduced version (ICC = 0.95–0.96; (Ruiz-Comellas et al., 2012)). In the present study, the questionnaire showed good internal consistency (Cronbach’s alpha = 0.80), confirming its reliability for assessing leisure-time physical activity in the analyzed sample.

The following Table 2 outlines the relationship between the study’s dimensions, objectives, and the standardized questionnaires employed (K-MPAI, MLTPAQ, and DPAA).

### 3.4. Data Analysis

Statistical analysis was performed using the Statistical Package for the Social Sciences (SPSS) software, version 25.0 (IBM Corp. Released, 2017). A bilateral significance level of α = 0.05 was adopted.

Initially, a descriptive analysis of individual items and questionnaire dimensions was conducted to characterize the data distribution and provide an overview of the study variables, addressing the first objective of identifying general trends in music performance anxiety (MPA), psychological determinants, and physical activity (PA).

Due to the sample size and the absence of normality in most variables, as indicated by the Kolmogorov–Smirnov test, nonparametric procedures were applied in the inferential analysis. Specifically, Mann–Whitney U and Kruskal–Wallis H tests were used to compare medians between independent groups (e.g., gender, academic level, or performance frequency), corresponding to the second objective of exploring intergroup differences.

Additionally, nonparametric bivariate correlations using Spearman’s coefficient were performed to examine the relationships between MPA, psychological resources, and PA, in line with the third objective aimed at identifying associations among the main constructs. Finally, multiple linear regression models were conducted to determine the predictive value of psychological and behavioral variables on MPA and PA outcomes, thereby addressing the fourth objective of the study and providing a comprehensive understanding of the factors influencing artistic performance and well-being.

## 4. Results

The normality analysis revealed that most variables did not follow a normal distribution, as indicated by the Kolmogorov–Smirnov test (*p* < 0.05). Consequently, nonparametric procedures were applied in the inferential analysis.

To facilitate interpretation and align the findings with the study objectives, results are presented according to the main research questions and statistical procedures applied.

### 4.1. Descriptive Analysis of MPA, Psychological Determinants, and Physical Activity

Regarding the levels of musical stage anxiety (MPA), considerable variability in scores was observed. The cognitive anxiety dimension presented a standard deviation of 3.43, higher than that of somatic anxiety (SD = 2.12), indicating greater heterogeneity in anticipatory thoughts compared to physiological symptoms. Likewise, self-confidence, as assessed by the DPAA, showed high dispersion (SD = 2.97), followed by the coping dimension (SD = 2.27), suggesting relevant interindividual differences in the psychological resources employed by the students.

### 4.2. Comparison by Gender, Course, and Musical Specialty

Descriptive statistics and *p*-values corresponding to comparisons by gender, course, and musical specialty are presented in Table 3. No statistically significant differences were detected in relation to gender or course. However, the music major was associated with significant differences in self-confidence (K-PAI and DPAA) and visualization (DPAA), with *p*-values < 0.05.

The comparative analysis by specialty, using the Kruskal–Wallis test, showed significant differences in self-confidence (K-MPAI) and visualization (DPAA). The results are presented in Table 4.

These results suggest that the type of major studied could influence the perception of self-confidence and the use of strategies such as visualization, which could be due to the stage demands of each discipline (such as singing, instrumental performance, or composition).

### 4.3. Correlations Among Psychological and Behavioral Variables

In the correlational analysis, a negative and significant relationship was identified between self-confidence and cognitive anxiety (ρ = −0.65, *p* < 0.001), indicating that students with higher confidence tend to experience fewer negative thoughts and less anticipatory stage worry. Additionally, positive correlations were observed between motivation, visualization, and coping, which supports the structural consistency of the DPAA questionnaire. In contrast, PA variables (METs, moderate and vigorous activity) did not show significant associations with the psychological dimensions assessed.

### 4.4. Mediation Analysis: Role of Self-Confidence

The CFA paragraph has been removed, as suggested by the reviewer, since psychometric validation was not an objective of the present study. The theoretical structure of the K-MPAI is now briefly mentioned in the Instrumentation section, citing Kenny (2009) and Zarza-Alzugaray et al. (2020).

Finally, a mediation model was estimated to analyze the role of self-confidence as a modulating variable in the relationship between motivation and cognitive stage anxiety. The results, summarized in Table 5, show a significant adverse indirect effect, which indicates that greater self-confidence partially attenuates the impact that motivation has on anxiety.

This pattern suggests that, although higher motivation may have been associated with higher levels of performance anxiety, possibly due to internal demands or performance pressure, the presence of self-confidence acts as a protective factor that mitigates this effect. The negative indirect influence confirmed the buffering role of self-confidence against anxious activation in musical performance contexts.

**Figure 1:**
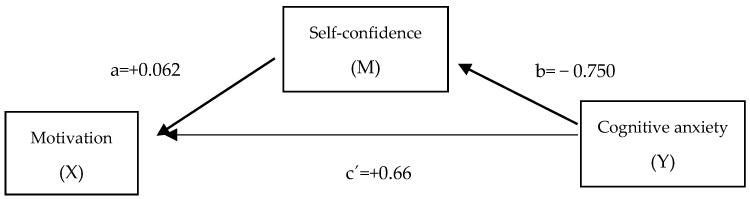
Model of the relationship between self-confidence, motivation, and cognitive anxiety.

The model is graphically represented in Figure 1:

## 5. Discussion

The high dispersion observed in cognitive versus somatic anxiety scores confirms the idea that anticipatory processes, such as worry, rumination, and fear of evaluation, constitute a fundamental core of MPA and explain a significant portion of its impact on academic and artistic performance. This finding aligns with Papageorgi et al. (2011), who emphasize the importance of cognitive components in the anxious experience, as well as with the work of Biasutti and Concina (2014) and Wilson and Roland (2002), which directly relate stage anxiety to difficulties in performance during public evaluations.

The negative correlation found between self-confidence and cognitive anxiety reinforces the protective role of self-efficacy described in the literature. Previous research has shown that self-efficacy constitutes a decisive factor in reducing MPA and coping more successfully with stage demands (Casanova et al., 2023; McPherson & McCormick, 2006; Ritchie & Williamon, 2011). In this sense, the results obtained align with the evidence indicating that personal confidence can attenuate the effects of self-demand and fear of negative judgment, two variables that typically amplify vulnerability to chronic stress (Butković et al., 2021; Patston & Osborne, 2016).

Although the sample size was limited, which may restrict the generalization of the results, the observed effect sizes corroborate the proposed relationships.

Visualization was positively associated with motivation and coping, exhibiting significant differences across musical specialties. These data support the findings described in previous research, which identify visualization as an effective cognitive strategy for anticipating performance, decreasing anxiety, and promoting better performance in musical evaluations (Braden et al., 2015; Brown & Palmer, 2013; Esplen & Hodnett, 1999; Finch & Moscovitch, 2016). The appearance of differences by specialty suggests that the application of this technique is not homogeneous and that it responds to the specific demands of each discipline’s stage, such as the greater degree of personal exposure in singing or the predominance of technical control in instrumental performance.

The estimated mediation model provides a relevant theoretical nuance. The results indicate that high motivation, if not accompanied by sufficient self-confidence, can intensify cognitive anxiety, consistent with the findings of Ou and Qin (2025) and McPherson and McCormick (2006). This does not contradict the reported benefits of intrinsic motivation and artistic enjoyment in reducing performance stress (Creech & Hallam, 2011; Ryan & Deci, 2000) but somewhat clarifies that their positive effect is mainly dependent on the prior consolidation of self-efficacy.

In this study, no significant associations were found between the practice of PA and the psychological variables evaluated. This result differs from that proposed by Biddle and Asare (2011), Craft and Perna (2004), Huang et al. (2024), Mikkelsen et al. (2017), and Wipfli et al. (2008), who have documented the benefits of PA in reducing anxiety and stress. A possible explanation lies in the fact that the questionnaire used collects the previous week’s practice and may not adequately reflect chronic habits or the temporal relationship with evaluative situations. Also, the impact of PA may depend on the context and accessibility to facilities and institutional programs that favor its integration into academic life (S. Donnelly et al., 2024; Lee et al., 2016; Reed, 2007; Sallis et al., 2016; Teuber et al., 2024).

Students’ entertainment habits also emerge as influential variables in emotional balance and coping with MPA. Excessive use of passive audiovisual media, such as television, social networks, or sedentary video games, has been associated with concentration problems, lower sleep quality, and increased emotional reactivity (Hale & Guan, 2015; Lemola et al., 2015; Twenge & Campbell, 2018). In contrast, active leisure activities such as active music listening, drawing, or video games that involve physical movement can induce positive emotional states and promote affective self-regulation (Benedek et al., 2014; Granic et al., 2014).

The absence of significant differences in MPA and psychological variables as a function of gender and academic year contrasts with Fernholz et al. (2019) and Papageorgi et al. (2011), who reported higher levels of anxiety in women or in those in early stages of training. A possible explanation for this is that the sample comes from a higher conservatory, where students tend to have more consolidated coping strategies and more stable career motivation.

## 6. Conclusions

The results of the study show that cognitive anxiety is the most relevant component of MPA in CSMC students. This dimension was negatively associated with self-confidence, suggesting that perceptions of personal efficacy act as a protective factor against anticipatory thoughts and excessive worry. Furthermore, the mediation model indicates that motivation can intensify anxiety when it is not accompanied by sufficient self-confidence, making this variable a central element in the management of performance in evaluative contexts.

Visualization was positively related to motivation and coping strategies and showed significant differences across musical specialties. This finding suggests that the application of this technique responds to the specific demands of each discipline and that its training should be adapted to the characteristics of each training area. The reinforcement of visualization as a teaching resource can reduce anxiety and prepare for evaluation situations, offering an intervention framework differentiated by specialty.

No significant associations were found between physical activity and the psychological variables analyzed, which contrasts with previous studies that have highlighted its benefits for emotional regulation. This absence of a relationship may be due to methodological limitations or to the situational nature of stage anxiety, underscoring the need to deepen this line of inquiry with longitudinal designs and specific protocols. MPA should be approached as a complex phenomenon in which self-confidence, motivation, and the use of psychological strategies play a determining role in students’ academic and artistic performance.

### Study Limitations

The sample size reduces the generalizability of the findings to other educational contexts or training levels. Furthermore, the cross-sectional design does not allow us to establish firm causal relationships between stage anxiety, self-confidence, motivation, and academic performance, so future longitudinal investigations would be necessary to confirm the observed patterns. 

Future research involving larger and more diverse samples could further validate these relationships and explore potential cultural or contextual variations in music performance anxiety.

## Figures and Tables

**Table 1:** Characteristics of the sample according to gender, course, and specialty.

Site	Specialty	Gender	n = Students	n = Total
Santa Cruz de Tenerife	Interpretation	Men	n = 12	n = 19
		Women	n = 7	
Gran Canaria	Interpreting	Men	n = 11	n = 37
		Women	n = 9	
		I prefer not to say	n = 1	
	Pedagogy	Men	n = 9	
		Women	n = 7	
Total				n = 56

**Table 2:** Relationship between the dimensions, objectives, and questions of the questionnaires.

Dimension	Subdimension	Objectives	Items
MSA	Cognitive anxiety	SO1, SO2, SO3	K-MPAI
	Somatic anxiety	SO1, SO2, SO3	K-MPAI
Psychological resources	Self-confidence	SO1, SO2, SO3, SO5	K-MPAI & DPAA
	Motivation	SO1, SO2, SO3, SO5	DPAA
	Coping	SO1, SO3, SO5	DPAA
	Attentional control	SO1, SO3, SO5	K-MPAI & DPAA
	Visualization	SO1, SO3, SO5	DPAA
Physical activity	Moderate activity	SO4	MLTPAQ
	Vigorous activity	SO4	MLTPAQ
	Total energy expenditure	SO4	MLTPAQ

**Table 3:** Comparison between groups by gender, grade, and musical specialty in the different dimensions analyzed.

Dimension	*p* Gender	*p* Course	*p* Specialty
Cognitive anxiety (K-MPAI)	0.98	0.87	0.14
Somatic anxiety (K-MPAI)	0.94	0.61	0.23
Self-confidence (K-MPAI)	0.45	0.60	0.02 *
Attentional control (K-MPAI)	0.32	0.84	0.19
Self-confidence (DPAA)	0.59	0.99	0.04 *
Motivation (DPAA)	0.79	0.58	0.21
Coping (DPAA)	0.36	0.45	0.14
Visualization (DPAA)	0.26	0.86	0.01 *
Attentional control (DPAA)	0.66	0.75	0.12
Moderate PA (MLTPAQ)	0.22	0.50	0.47
Vigorous PA (MLTPAQ)	0.19	0.69	0.37
MET total (MLTPAQ)	0.36	0.78	0.39

Legend: * *p* < 0.05 indicates a statistically significant difference.

**Table 4:** Comparisons by musical specialty on key dimensions of Self-Confidence and Visualization.

Dimension	Test	Statistician H	Specialty		*p*-Value
Self-confidence (K-MPAI)	Kruskal–Wallis	10.13	1	3.47	0.018
			2	3.19	
Visualization (DPAA)	Kruskal–Wallis	11.47			0.012

Note. 1 = Pedagogy. 2 = Interpretation.

**Table 5:** Mediation model: Self-confidence as a modulating variable between motivation and cognitive anxiety.

Relation	Coefficient
Motivation—Self-confidence (a)	+0.062
Self-confidence—Anxiety (b)	−0.750
Motivation—Direct anxiety (c′)	+0.660
Indirect effect (a × b)	−0.046

## Data Availability

The data supporting the findings of this study are not publicly available due to privacy and ethical restrictions related to the protection of participants’ personal information.

## Abbreviations

The following abbreviations are used in this manuscript:

MPA	Musical stage anxiety
PA	Physical activity
